# The interaction between blood lipids and ASCVD increases the risk of DKD: a nonlinear relationship transforms into a linear relationship, a cross-sectional study

**DOI:** 10.3389/fendo.2025.1652396

**Published:** 2025-08-20

**Authors:** Huan Li, Yulu Shi, Hui Zhang, Jie Han, Xiaoping Zhang, ZiJie Liu

**Affiliations:** ^1^ First Affiliated Hospital, Kunming Medical University, Kunming, China; ^2^ Yunnan Key Laboratory of Laboratory Medicine, Kunming, China; ^3^ Department of Clinical Laboratory, the First Affiliated Hospital of Kunming Medical University, Kunming, China; ^4^ Yunnan Province Clinical Research Center for Laboratory Medicine, Kunming, China

**Keywords:** diabetes mellitus type 2, ASCVD, diabetic kidney disease, lipids, LDL-C, TC

## Abstract

**Background:**

Atherosclerotic cardiovascular disease (ASCVD) and diabetic kidney disease (DKD) are interconnected vascular complications in diabetes, with dyslipidemia playing a key role. The modifying effect of ASCVD on the lipid-DKD relationship in diabetic patients without lipid-lowering treatment remains unclear.

**Methods:**

This retrospective study included 26,476 type 2 diabetic patients without lipid-lowering therapy. Associations between lipids (LDL-C, TC, TG, HDL-C) and DKD risk were analyzed using regression and restricted cubic spline (RCS) curves analysis. Both multiplicative and additive interactions between lipids and ASCVD were assessed.

**Results:**

HDL-C showed a significant linear association with DKD. RCS analyses revealed distinct patterns based on ASCVD status: significant threshold effects for LDL-C (2.68 mmol/L), TC (4.29 mmol/L), TG (2.48 mmol/L), and HDL-C (1.64 mmol/L) on DKD risk were observed only in diabetic patients without ASCVD. No significant nonlinear threshold effects were found for LDL-C, TC, HDL-C on DKD risk in diabetic patients with ASCVD. LDL-C and TC showed continuous increases in DKD risk without a discernible safe threshold in diabetic patients with ASCVD. Crucially, a strong synergistic interaction existed between ASCVD and both TC (RERI=7.46, AP=0.25, SI=1.34) and LDL-C (RERI=9.91, AP=0.27, SI=1.38), significantly amplifying their adverse effects on renal injury.

**Conclusion:**

ASCVD amplifies the detrimental renal effects of TC and LDL-C and eliminates protective lipid thresholds in diabetic patients. Consequently, lipid management in diabetic patients should be individualized: strict control of TC and LDL-C is prioritized for those with ASCVD, while consideration of lipid threshold effects is key for those without ASCVD.

## 1 Introduction

Diabetes mellitus causes significant harm through both macrovascular and microvascular complications (1, 2). A key macrovascular complication is ASCVD, which affects approximately 50% of individuals with diabetes. DKD, the primary microvascular complication affecting 30–40% of diabetic patients, significantly contributes to end-stage renal failure (3). These complications are major mortality factors in diabetes and create a vicious cycle due to shared pathological mechanisms such as vascular inflammation and endothelial dysfunction. ASCVD accelerates renal injury, whereas DKD exacerbates cardiovascular outcomes (4). The recently introduced “cardiovascular-kidney-metabolic (CKM) syndrome” highlights how cardiac, renal, and metabolic disorders form a pathological loop driven by oxidative stress, chronic inflammation, and neuroendocrine activation. This results in a lethal cycle known as the “metabolism-heart-kidney” triple hit (5, 6). Disrupting this cycle depends on identifying intervention targets with cross-organ protective effects.

Dyslipidemia is central to CKM syndrome due to its vascular toxicity (7), but lipid metabolism in CKM remains poorly defined. LDL-C drives atherosclerosis (8) and correlates with renal dysfunction in diabetes. It may directly damage kidney via glomerular deposition, activating TGF-βand promoting fibrosis (9, 10). However, Mendelian randomization suggests no direct causal link to eGFR decline, implying LDL-C nephrotoxicity requires specific pathological contexts (11). HDL-C inversely correlates with UACR (12), but its cardiorenal benefits may be reduced in diabetes or chronic inflammation due to altered functionality (13, 14). Elevated TG and TC contribute to cardiovascular risk and DKD progression (15, 16).Current lipid guidelines prioritize cardiovascular outcomes and LDL-C goals, lacking strategies for renal protection (17). Crucially, most studies fail to account for baseline lipid-lowering therapy. lipid-lowering therapy modify lipid profiles and renal inflammation, confounding observational studies and limiting the generalizability of findings on native lipids’ renal effects. Therefore, investigating lipid-DKD relationships in diabetic patients without lipid-lowering therapy is essential.

ASCVD may dynamically influence the lipid-DKD risk relationship by modifying the systemic inflammatory microenvironment and remodeling lipoprotein metabolism. However, the impact of diabetes accompanied by ASCVD on the progression of DKD remains insufficiently explored, and interactions among diabetic complications are inadequately considered. This gap hinders the establishment of customized lipid management.

This study aims to (1) elucidate the dose–risk relationships between specific lipid components and the risk of DKD and (2) assess whether the coexistence of ASCVD affects these associations. These findings provide evidence for personalized lipid management, facilitating a shift beyond isolated organ-focused treatments toward comprehensive strategies.

## 2 Method

### 2.1 Study population

This study, designed as a retrospective cross-sectional analysis, examined the medical records of T2DM patients at the First Affiliated Hospital of Kunming Medical University between January 1, 2020, and December 31, 2024.

The inclusion criteria:

1. age ≥18 years with intact cognitive capacity;
2. meeting the diagnostic criteria of the Chinese Guidelines for Type 2 Diabetes Prevention and Treatment (2020);
3. availability of complete clinical datasets;

The exclusion criteria:

1. type 1 diabetes mellitus (T1DM) or other specific diabetes types;
2. history of malignancy;
3. acute diabetic complications (diabetic ketoacidosis or hyperosmolar hyperglycemic state);
4. prior lipid-lowering therapy (statins, fibrates, PCSK9 inhibitors, or related agents).

### 2.2 Ethics statement

This study utilized anonymized clinical and laboratory data from hospital records. The research protocol was approved by the Ethics Committee at the First Affiliated Hospital of Kunming Medical University[Approval No. (2022) Lunshen 61]. Given that the research exclusively involved deidentified historical data, the ethics committee granted a waiver of informed consent. During data extraction and analysis, all personally identifiable information (such as names and ID numbers) was removed, and only study-relevant anonymized variables were retained. Data access was restricted to authorized research personnel, with encrypted storage on secured servers and regular backups ensuring information security. All procedures complied with the Personal Information Protection Law and institutional data governance policies to safeguard patient privacy and rights. De-identified data supporting this study are available upon reasonable request to the corresponding author, subject to approval by the Ethics Committee of the First Affiliated Hospital of Kunming Medical University. Data sharing complies with institutional governance policies and China’s Personal Information Protection Law.

### 2.3 Data collection and variable definitions

A total of 26,476 patients with T2DM were included, including 24,400 with T2DM only, 268 with T2DM and ASCVD, 692 with T2DM, ASCVD and DKD, and 1,116 with T2DM and DKD.

Data collection included:

1. Demographic characteristics: age and sex;
2. Blood pressure: systolic and diastolic measurements;
3. Metabolic indices: Fasting blood glucose (FBG), total cholesterol (TC), triglyceride (TG), high-density lipoprotein cholesterol (HDL-C), and low-density lipoprotein cholesterol (LDL-C) levels.
4. Renal function parameters: Serum creatinine (Scr), uric acid (UA), urea, N-acetyl-β-D-glucosaminidase-to-creatinine ratio (NAG/Cr), urinary glucose (UGlu), urinary microalbumin (UMA), urinary total protein (UTP), urinary creatinine (Ucr), N-acetyl-β-D-glucosaminidase (NAG), the urine protein-to-creatinine ratio (UPCR), and random urine glucose (RUG).

#### 2.3.1 Exposure variables

Four lipid indicators—low-density lipoprotein cholesterol (LDL-C), high-density lipoprotein cholesterol (HDL-C), triglyceride (TG), and total cholesterol (TC)—were included as continuous exposure variables. Lipid levels were modeled as continuous measures.

#### 2.3.2 Outcome variables

DKD was defined as the primary outcome according to the Chinese Guidelines for the Prevention and Treatment of Diabetic Kidney Disease (2024).

#### 2.3.3 Subgroup variables

ASCVD was employed as a subgroup variable, with diagnoses by the Chinese Guidelines for the Diagnosis and Management of Patients with Chronic Coronary Syndrome (2024).

### 2.4 Statistical analysis

#### 2.4.1 Data imputation

The percentages of missing data were as follows: diabetes group (urea: 17.94%, creatinine: 9.62%, uric acid: 3.83%, blood pressure: 12.95%) and DKD group (urea: 0.65%, creatinine: 0.65%, uric acid: 1.09%, blood pressure: 19.80%). Missing values were imputed via iterative random forest multiple imputation. An initially complete dataset was generated through random value assignment. The variables were subsequently imputed sequentially by treating the current missing variable as the target and all other variables as features. A random forest model was trained on observed data via bootstrap sampling to generate multiple decision trees, with random feature subset selection at each node.

Continuous missing values were replaced with the mean prediction across trees, whereas categorical missing values were sampled from the predictive probability distribution. This iterative process cycles through all the variables five times until convergence is achieved (defined as minimal changes in imputed values). To account for imputation uncertainty, five independent imputation cycles were performed with randomized initial values, generating distinct complete datasets.

#### 2.4.2 Linear analysis

Normally distributed continuous variables are expressed as means ± standard deviations, and those with nonnormal distributions are presented as medians (interquartile ranges, IQRs). The initial demographic and clinical features were analyzed via appropriate statistical methods: Chi-square tests (categorical), Student’s t tests (normal continuous), and Mann-Whitney U tests (non-normal continuous).

A binary logistic regression model was formulated, using DKD diagnosis as the dependent variable and including the specified independent variables: TG, TC, HDL-C, LDL-C, FBG, UA, BP, age, and sex. Significant predictors through univariate analysis incorporated into the multivariate analysis, with adjustments made for age, sex, and blood pressure as potential confounders.

#### 2.4.3 RCS and threshold effect analysis

Nonlinear relationships were analyzed by RCS. This method utilizes piecewise cubic polynomials joined at predefined knots, with linear constraints applied to the tails (first and last segments) to ensure stable extrapolation. Knots were positioned at the 5th, 35th, 65th, and 95th percentiles of lipid distributions. Nonlinearity was formally evaluated via likelihood ratio tests comparing the RCS model against a linear model, adjusting for age, sex, and blood pressure. When nonlinear relationships were identified, inflection points were determined through piecewise regression modeling. The statistical significance of odds ratio (OR) differences across identified thresholds was subsequently calculated.

#### 2.4.4 Multiplicative and additive interactions

Multiple interactions were assessed by incorporating product terms between lipid indices and ASCVD. The significance of multiplicative interactions was assessed via likelihood ratio tests on nested models. Additive interactions were analyzed through multivariable logistic regression after adjusting for age, sex, and blood pressure. Three interaction indices were calculated: the relative excess risk due to interaction (RERI), the attributable proportion due to interaction (AP), and the synergy index (SI). The RERI quantifies excess risk from coexposures (RERI > 0 indicates a positive interaction). AP represents the proportion of disease risk attributable to the interaction. The SI measures the synergistic effect intensity (SI > 1 indicates synergy between factors).

(5) Statistical analyses were conducted with R 4.3.1. Packages used included “bloom”, “segmented”, “interaction”, and “EPIR”. We defined significance as *p*<0.05 (two-tailed).

## 3 Results

### 3.1 Characteristics of the participants

Diabetic patients with DKD were significantly younger than patients with DM alone more likely to be male. DKD patients presented elevated SBP, DBP, UACR, UMA, NAG/Cr, Scr, UA, urea, UTP, UPCR, RUG and NAG levels. Hypertension prevalence, defined as SBP ≥140 mmHg or DBP ≥90 mmHg, was significantly higher in patients with DKD (78.3%) than in those with DM alone (52.6%).

Conversely, the eGFR was significantly reduced. Lipid profiles demonstrated significant differences between the groups: TG and LDL-C were elevated in the DKD group, whereas HDL-C was reduced. TC did not differ significantly (**Table 1**).

**Table 1:** Characteristics of the participants.

Variables	DM (n=24675)	DKD (n=1801)	z/*x^2^*	*p*
Age(year)^b^	59.82 ± 7.66	52.94 ± 10.29	2.21	0.034
Gender, n(%)^a^			4.25	0.039
male	14456.00 (58.59)	1195.00(66.35)		
female	10219.00 (41.41)	606.00(33.65)		
SBP(mmHg^b^)	123.92(118.60,130.00)	132.01(128.00,136.80)	-32.26	<.001
DBP(mmHg^b^)	77.33(74.00,80.90)	81.41(78.00,84.20)	-24.23	<.001
UACR^b^	11.65(5.60,32.50)	98.58(40.50,449.60)	-47.64	<.001
UMA(mg/L)^b^	12.40(5.60,33.80)	78.40(29.90,362.10)	-42.21	<.001
Ucr(umol/L)^b^	9022.00(5925.00,13130.00)	6647.00(4560.50,9743.50)	-19.63	<.001
UTP(g/L)^b^	0.09(0.10,0.20)	0.22(0.1,0.6)	-28.28	<.001
NAG/Cr(U/g/Cre)^b^	11.91(7.50,19.80)	18.35(11.3,29.5)	-22.32	<.001
RUG(mmol/L)^b^	2.11(0.50,82.10)	11.87(1.0,93.1)	-9.70	<.001
NAG(U/L)^b^	12.20(7.50,19.80)	13.50(8.4,22.0)	-6.18	<.001
UA(umol/L)^b^	343.50(285.20,405.40)	368.50(301.60,441.40)	-10.06	<.001
Urea(umol/L)^b^	5.45(4.60,6.40)	6.30(5.00,8.00)	-18.51	<.001
Scr(umol/L)^b^	74.30(63.00,86.90)	86.69(70.50,109.50)	-21.64	<.001
UPCR(mg/g Cre)^b^	85.61(54.80,169.10)	270.96(130.20,766.20)	-39.07	<.001
FBG(mmol/L)^b^	7.29(5.90,9.30)	7.31(5.05,9.90)	-0.78	0.438
TG(mmol/L)^b^	1.64(1.10,2.50)	1.80(1.20,2.70)	-5.85	<.001
HDL-C(mmol/L)^b^	1.06(0.90,1.30)	0.97(0.80,1.20)	-12.07	<.001
TC(mmol/L)^b^	4.43(3.70,5.20)	4.38(3.60,5.30)	-0.89	0.376
LDL-C(mmol/L)^b^	2.63(2.00,3.20)	2.54(1.80,3.30)	-2.99	0.003
eGFR(ml/min/1.73 m²)^b^	91.58(77.40,102.30)	76.05 (57.06, 94.43)	-23.62	0.006

a chi-square test.

b Mann–Whitney U test.

### 3.2 Linear relationship between blood lipids and DKD risk

Using DKD as the dependent variable, logistic models tested the independent association of lipids with DKD risk (**Tables 2**, **3**). Univariate analysis revealed significant positive associations between DKD risk and sex, age, TG, SBP, and DBP. In contrast, HDL-C demonstrated a negative association, whereas LDL-C showed no significant relationship. Following multivariate adjustment for significant univariate predictors, sex, age, UA, HDL-C, SBP, and DBP remained independently associated with DKD risk. TG lost significance in the multivariate model, indicating potential confounding by other variables.

**Table 2:** Univariate analysis.

Variables	β	S.E	Z	*P*	OR (95%CI)
female					1.00 (Reference)
male	0.33	0.05	6.45	<.001	1.39 (1.26 ~ 1.54)
Age	0.02	0.00	12.78	<.001	1.03 (1.02 ~ 1.03)
UA(mmol/L)	0.01	0.00	10.17	<.001	1.01 (1.01 ~ 1.01)
FBG(mmol/L)	-0.00	0.01	-0.00	0.998	1.00 (0.99 ~ 1.01)
TG(mmol/L)	0.03	0.01	3.93	<.001	1.04 (1.02 ~ 1.05)
HDL-C(mmol/L)	-0.97	0.09	-10.99	<.001	0.38 (0.32 ~ 0.45)
LDL-C(mmol/L)	-0.03	0.03	-1.30	0.194	0.97 (0.92 ~ 1.02)
SBP(mmHg)	0.04	0.00	24.58	<.001	1.04 (1.04 ~ 1.05)
DBP(mmHg)	0.04	0.00	16.35	<.001	1.05 (1.04 ~ 1.05)

**Table 3:** Multivariate analysis.

Variables	β	S.E	Z	*P*	OR (95%CI)
Intercept	-8.90	0.32	-27.90	<.001	0.00 (0.00 ~ 0.00)
female					1.00 (Reference)
male	0.25	0.06	4.57	<.001	1.29 (1.16 ~ 1.44)
Age	0.03	0.00	11.75	<.001	1.03 (1.02 ~ 1.03)
UA(mmol/L)	0.01	0.00	6.47	<.001	1.01 (1.01 ~ 1.01)
TG(mmol/L)	0.00	0.01	0.32	0.753	1.00 (0.98 ~ 1.02)
HDL(mmol/L)	-0.87	0.10	-8.58	<.001	0.42 (0.34 ~ 0.51)
SBP(mmHg)	0.03	0.00	14.43	<.001	1.03 (1.03 ~ 1.04)
DBP(mmHg)	0.01	0.00	3.04	0.002	1.01 (1.01 ~ 1.02)

### 3.3 Dose–response relationships and threshold effect analyses of blood lipids and DKD risk

**Figure 1:**
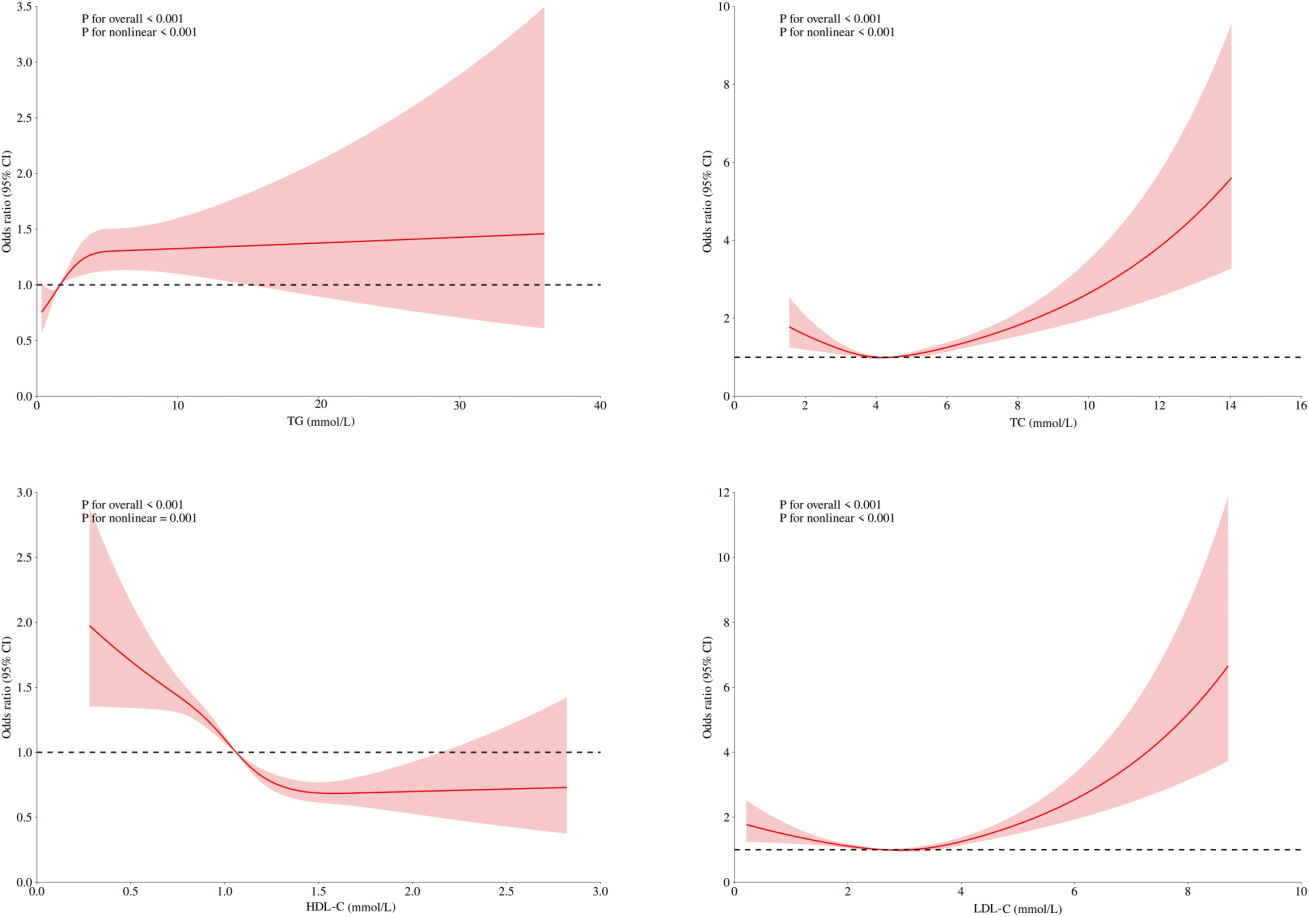
RCSs for the dose–response relationship between blood lipids and the risk of DKD. RCS of LDL-C, HDL-C, TC, and TG revealed a nonlinear relationship with DKD risk, adjusted for sex, age, blood pressure, fasting blood glucose, and UA.

After adjustment for age, sex, and blood pressure, dose–response relationships between lipid parameters and DKD risk were modeled via RCS (**Figure 1**). Significant nonlinear associations were found for LDL-C, HDL-C, TG and TC (*p*< 0.001). Segmented linear regression modeling revealed statistically significant threshold effects for all four lipid parameters (**Table 4**).

**Table 4:** Threshold effect analysis of blood lipids and DKD risk.

Diabetic kidney disease	OR (95%CI)	*P* value
LDL-C(mmol/L):		
Model 1 Fitting model by standard linear regression	0.98 (0.94 - 1.04)	0.563
Model 2 Fitting model by two-piecewise linear regression		
Inflection point of LDL-C	2.68	
<2.68	0.72 (0.63 - 0.82)	<.001
≥2.68	1.25 (1.14 - 1.37)	<.001
P for likelihood test		<.001
HDL-C(mmol/L):		
Model 1 Fitting model by standard linear regression	0.38 (0.32 - 0.46)	<.001
Model 2 Fitting model by two-piecewise linear regression		
Inflection point of HDL-C	1.64	
<1.64	0.31 (0.25 - 0.39)	<.001
≥1.64	2.21 (0.84 - 5.82)	0.110
*p* for likelihood test		<.001
TG(mmol/L):		
Model 1 Fitting model by standard linear regression	1.05 (1.03 - 1.06)	<.001
Model 2 Fitting model by two-piecewise linear regression		
Inflection point of TG	2.48	
<2.48	1.36 (1.21 - 1.52)	<.001
≥2.48	1.01 (0.98 - 1.04)	0.451
*p* for likelihood test		<.001
TC( mmol/L):		
Model 1 Fitting model by standard linear regression	1.05 (1.01 - 1.09)	0.027
Model 2 Fitting model by two-piecewise linear regression		
Inflection point of TC	4.29	
<4.29	0.80 (0.70 - 0.90)	<.001
≥4.29	1.17 (1.10 - 1.23)	<.001
*p* for likelihood test		<.001

Distinct thresholds were identified for each lipid parameter (**Figure 1**). LDL-C (2.68 mmol/L) and TC (4.29 mmol/L) demonstrated inverse associations with DKD risk below their respective thresholds; however, concentrations that exceeded these values significantly heightened the risk (P<0.001). For HDL-C (1.64 mmol/L), significant protective effects against DKD were observed below the threshold, although no significant association was detected at higher concentrations (*p*=0.12). TG exhibited a distinct pattern: levels below 2.48 mmol/L were associated with progressively elevated DKD risk (*p*<0.001), whereas no additional risk increase occurred beyond this threshold (*p*=0.84).

### 3.4 Dose–response relationships and threshold effect analyses of blood lipid components and DKD risk in diabetic patients with ASCVD

**Figure 2:**
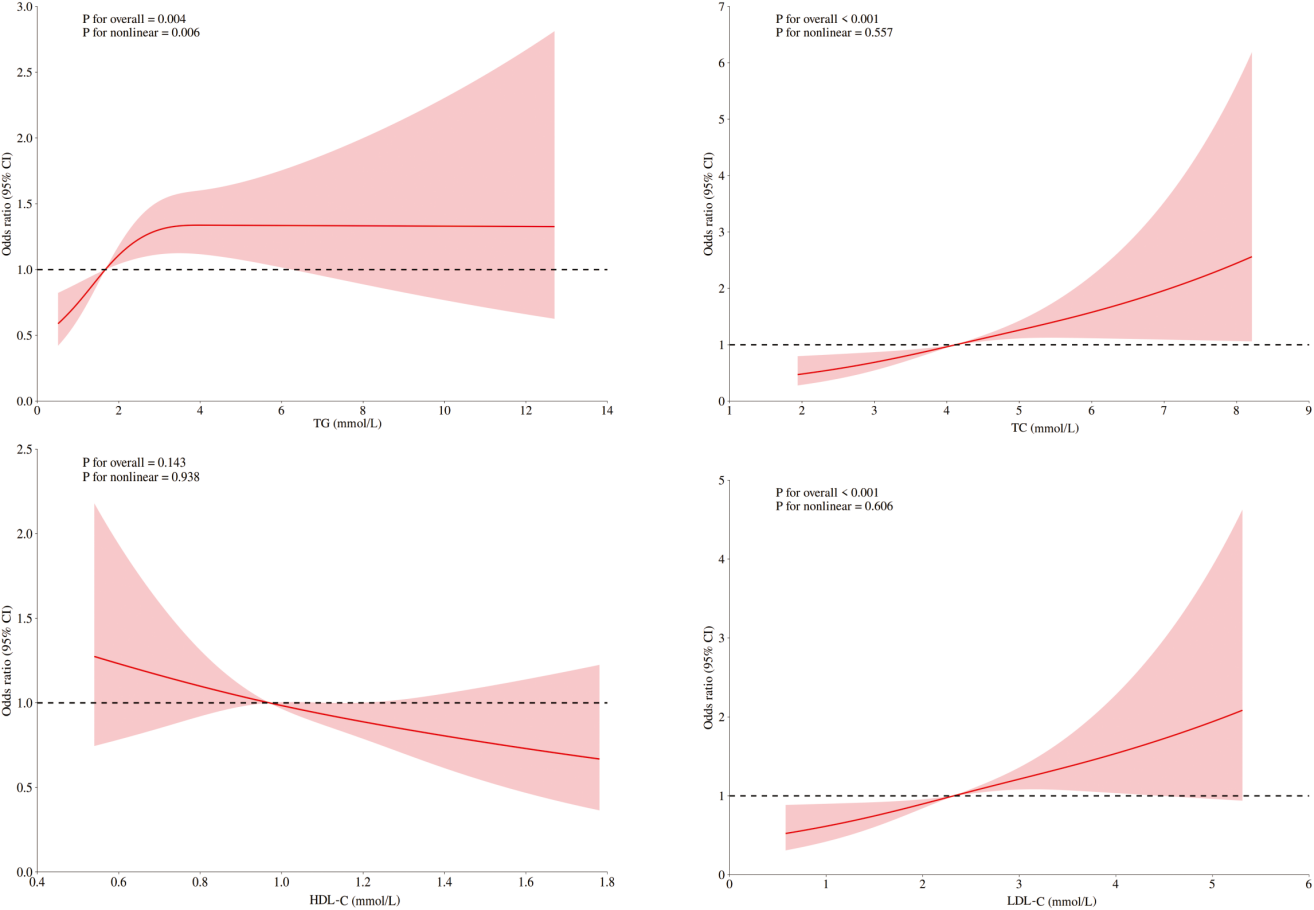
RCSs of blood lipids and DKD risk in diabetic patients with ASCVD. RCS of the nonlinear relationships of LDL-C, HDL-C, TC, and TG with DKD risk in diabetic patients with ASCVD adjusted for sex, age, blood pressure, fasting blood glucose, and UA.

**Figure 3:**
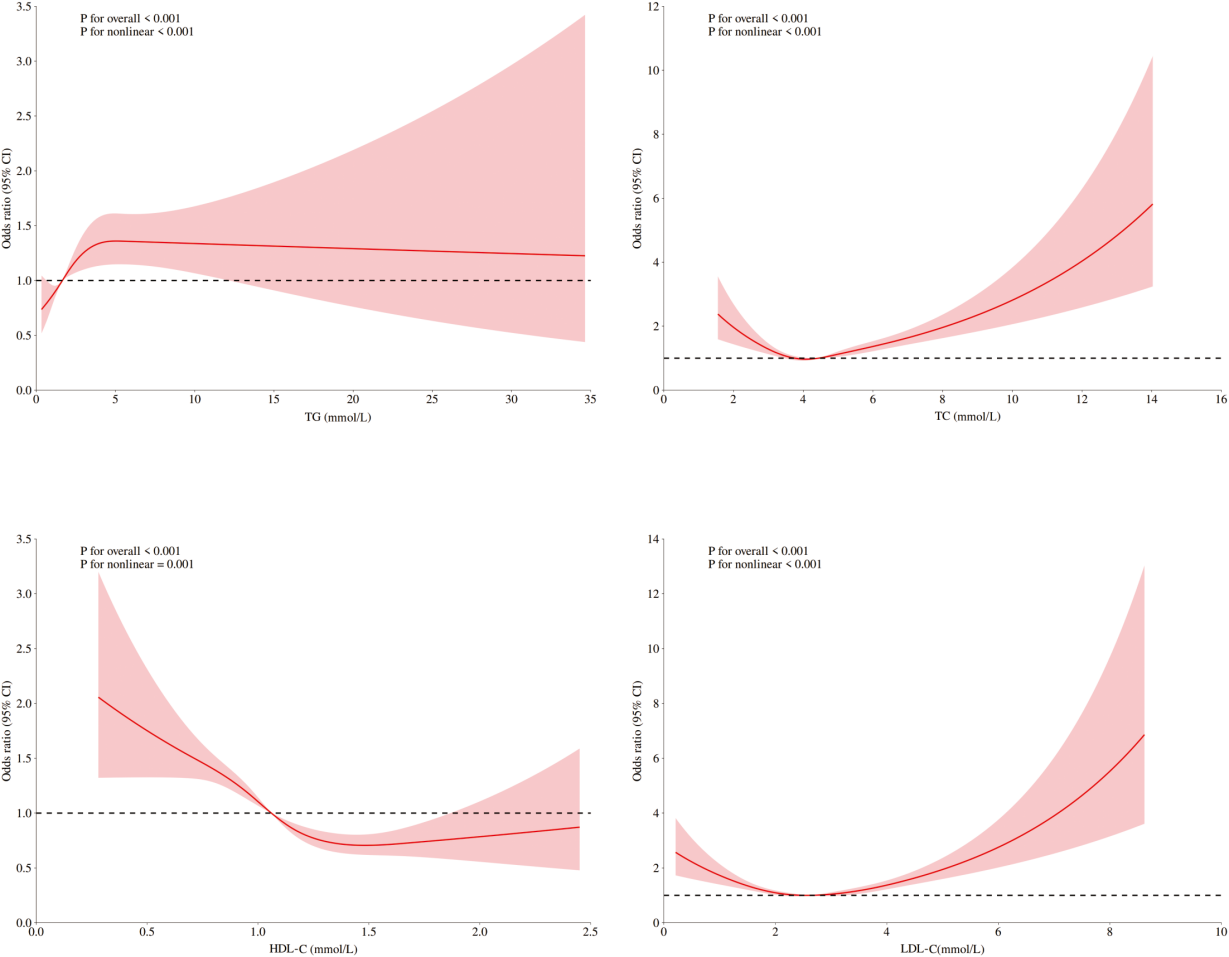
RCSs of blood lipids and DKD risk in diabetic patients without ASCVD. RCS of the nonlinear relationships of LDL-C, HDL-C, TC, and TG with DKD risk indiabetic patients without ASCVD adjusted for sex, age, blood pressure, fasting blood glucose, and UA.

Among ASCVD patients, HDL-C showed no significant association with DKD risk, whereas LDL-C and TC were linearly related. Only TG demonstrated significant nonlinearity with respect to the risk of DKD. Conversely, all four lipids exhibited significant nonlinear associations and threshold effects in non-ASCVD patients (**Figures 2**, **3**). Threshold effect analysis revealed the following lipid thresholds: LDL-C at 2.68 mmol/L, HDL-C at 1.64 mmol/L, TG at 2.48 mmol/L, and total cholesterol (TC) at 4.29 mmol/L. (**Table 5**). These findings indicate that ASCVD significantly affects lipid-DKD risk relationships in diabetic patients.

**Table 5:** Threshold effect analysis of blood lipids and DKD risk in diabetic patients without ASCVD.

Diabetic kidney disease	OR (95%CI)	*P* value
LDL-C(mmol/L):		
Model 1 Fitting model by standard linear regression	1.05 (0.98 - 1.12)	0.147
Model 2 Fitting model by two-piecewise linear regression		
Inflection point of LDL-C	2.46	
<2.68	0.68 (0.56 - 0.83)	<.001
≥2.68	1.29 (1.17 - 1.42)	<.001
*p* for likelihood test		<.001
HDL-C(mmol/L):		
Model 1 Fitting model by standard linear regression	0.46 (0.36 - 0.57)	<.001
Model 2 Fitting model by two-piecewise linear regression		
Inflection point of HDL-C	1.63	
<1.64	0.34 (0.26 - 0.45)	<.001
≥1.64	3.51 (1.30 - 9.46)	0.013
*p* for likelihood test		<.001
TG(mmol/L):		
Model 1 Fitting model by standard linear regression	1.04 (1.02 - 1.07)	<.001
Model 2 Fitting model by two-piecewise linear regression		
Inflection point of TG	2.69	
<2.48	1.36 (1.19 - 1.54)	<.001
≥2.48	1.00 (0.97 - 1.03)	0.948
*p* for likelihood test		<.001
TC(mmol/L):		
Model 1 Fitting model by standard linear regression	1.09 (1.04 - 1.15)	<.001
Model 2 Fitting model by two-piecewise linear regression		
Inflection point of TC	4.20	
<4.29	0.81 (0.68 - 0.96)	0.017
≥4.29	1.19 (1.13 - 1.27)	<.001
*p* for likelihood test		<.001

### 3.5 The impact of ASCVD and blood lipid interactions on the risk of DKD

A significant multiplicative interaction between ASCVD and blood lipid levels was identified with respect to DKD risk. TC was positively associated with DKD risk in patients without ASCVD (OR=1.095, 95% CI: 1.044–1.146) and was strongly associated with DKD risk in those with ASCVD (OR=1.293, 95% CI: 1.133–1.483). LDL-C was significantly linked to DKD risk only in patients with ASCVD, demonstrating a notable multiplicative interaction. In contrast, no significant multiplicative interaction was detected between ASCVD and either TG or HDL-C (**Table 6**).

**Table 6:** Association of blood lipids with DKD Risk by ASCVD.

Blood lipid component	OR	95%CI Lower Limit	95%CI Upper Limit	Wald value	*p* value	*p* value of multiplicative interaction
TG						0.5256
without ASCVD	1.044	1.021	1.065	3.995	<0.001	
With ASCVD	1.046	0.976	1.137	1.154	0.249	
TC						0.003571
without ASCVD	1.095	1.044	1.146	3.796	<0.001	
With ASCVD	1.293	1.133	1.483	3.749	<0.001	
HDL-C						0.3323
without ASCVD	0.456	0.364	0.569	-6.868	<0.001	
With ASCVD	0.634	0.365	1.102	-1.621	0.105	
LDL-C						0.001269
without ASCVD	1.048	0.983	1.116	1.451	0.147	
with ASCVD	1.341	1.143	1.581	3.553	<0.001	

ASCVD and TC, LDL-C showed synergistic interaction by additive analysis in increasing DKD risk (**Table 7**). When ASCVD coexists with elevated TC, the DKD risk significantly exceeds the individual effects. The interaction between LDL-C and ASCVD was even stronger, with the interaction accounting for 27% of the combined effect of LDL-C and ASCVD on DKD risk. In contrast, no statistically significant additive interaction was detected between ASCVD and either TG or HDL-C (both *p*>0.05). These results demonstrated that the interactions between ASCVD and TC or LDL-C significantly amplified the DKD risk associated with these lipid parameters.

**Table 7:** Effects of the additive interaction of lipids and ASCVD on the risk of DKD.

	RERI (95%CI)	AP (95%CI)	SI (95%CI)
TG	3.25(-0.25,6.75)	0.06(-0.01,0.14)	1.07(0.99,1.16)
TC	7.46(5.49,9.42)***	0.25(0.15,0.34)***	1.34(1.17,1.54)***
HDL-C	-13.62(-33.58,6.35)	-0.61(-1.47,0.26)	0.61(0.35,1.06)
LDL-C	9.91(6.75,13.07)***	0.27(0.15,0.38)***	1.38(1.17,1.63)***

***p value <0.001.

Although the multiplicative interaction of TC,LDL-C and ASCVD was only significant in men and the elderly, the additive interaction confirmed that TC,LDL-C and ASCVD increased the risk of DKD(**Supplementary Tables 1**, **2**). ASCVD combined with high TC or LDL-C leads to a synergistic increase in the risk of DKD.

## 4 Discussion

The study revealed no significant association between HDL-C and DKD risk in diabetic patients with ASCVD. LDL-C and TC levels were linearly related to DKD risk, whereas TG levels were significantly nonlinearly related. In diabetic patients without ASCVD, all four lipids were significantly associated with DKD risk, with significant threshold effects. Interaction analysis indicated that ASCVD significantly increased the DKD risk caused by TC and LDL-C. These findings support lipid management strategies for diabetic patients on the basis of ASCVD status. As a cross-sectional study, our findings identify associations but cannot establish causality. Relationships between lipids, ASCVD, and DKD require validation in longitudinal cohorts. Residual confounding like unmeasured genetic or dietary factors may also influence results.

By excluding lipid-lowering drug users, this study significantly reduced pharmacological confounding, enabling direct assessment of dyslipidemia-DKD associations. Second, RCS and threshold analysis revealed nonlinear lipid–DKD risk associations, enabling early high-risk identification. Importantly, ASCVD was shown to modify the relationship between lipids and DKD risk, suggesting a mechanistic link between systemic inflammation and lipid metabolism disorders.

These findings establish ASCVD as a key indicator for DKD risk stratification, guiding clinical approaches. For diabetic patients without ASCVD, maintaining LDL-C and TC levels near the identified threshold while avoiding extremes is recommended. Cardiovascular protection is paramount in diabetic patients with ASCVD. Guideline-directed lipid targets (e.g., LDL-C <1.8 mmol/L) should be strictly adhered to despite renal risk considerations, as ASCVD exacerbates lipid pathways contributing to DKD. TG management requires intervention below the threshold to reduce cumulative renal damage, transitioning to multifactorial risk control when the threshold is exceeded. HDL-C interventions should focus on enhancing function rather than merely increasing its concentration. Overall, this evidence shifts clinical practice from numerical lipid targets toward risk-stratified organ protection strategies, developing lipid management for DKD patients with coexisting ASCVD.

These four lipids revealed statistically significant relationships when analyzed via RCS modeling. This discrepancy likely stems from their nonlinear associations with DKD risk, which linear methods cannot detect. The RCS analysis overcomes this limitation by flexibly modeling threshold effects and nonlinear exposure–response relationships, thus revealing clinically meaningful links between blood lipids and DKD risk.

In diabetic patients without ASCVD, blood lipids may reflect the pathological mechanisms underlying DKD risk in a nonlinear manner. RCS analysis revealed that LDL-C and TC levels below certain thresholds were linked to increased DKD risk. This challenges the conventional “lower is better” rules, suggesting complex biological interactions: 1) Low LDL-C and TC may indicate systemic metabolic dysregulation, such as chronic inflammation or complications, serving as markers of disease severity rather than direct pathogenic factors. 2) Excessive lipid reduction could impair renal function by disrupting cellular membrane integrity or steroidogenesis. Conversely, elevated LDL-C and TC beyond thresholds can exceed renal metabolic capacity, leading to kidney injury via oxidative stress pathways.

These nonlinear relationships are supported by recent studies, including a cross-sectional analysis at Zhejiang University’s Affiliated Jinhua Hospital showing increased DKD risk at LDL-C <2.97 mmol/L (18) and Shanghai community data identifying TG (1.90 mmol/L) and HDL-C (1 mmol/L) thresholds (19). Importantly, these statistical inflection points represent population-level estimates rather than individualized targets. Significant interpatient variability in metabolic status, complications, and medications necessitates comprehensive clinical evaluation. Therefore, elevated DKD risk at low LDL-C levels should be interpreted cautiously, likely reflecting malnutrition or advanced comorbidities rather than direct LDL-C toxicity.

Limitations in the protective role of HDL-C align with European observations of the “HDL-C paradox” (20, 21). HDL-C showed significant protective effects against DKD only at concentrations below 1.64 mmol/L, with this association plateauing beyond that threshold. This attenuation may stem from insulin resistance and impaired particle functionality, which reduce cholesterol efflux capacity (22, 23). The threshold effect for TG likely reflects the free fatty acid metabolic balance. Below this threshold, moderate TG elevation may increase DKD risk through direct lipotoxic damage to glomerular endothelial cells. The above-threshold TG level was not significantly associated with DKD risk, possibly because severe insulin resistance in hypertriglyceridemic patients obscures the independent effects of TG, as hyperglycemia becomes the primary cause of renal damage (24, 25). These findings partially support Mendelian randomization studies establishing causal links in HDL-C, TG, and DKD risk (11), although complex pathophysiology of diabetes cannot be attributed exclusively to genetic influences.

In diabetic patients with ASCVD, LDL-C and TC were linearly associated with DKD risk, suggesting that ASCVD alters blood lipid-DKD risk relationships. The lipid thresholds in patients without ASCVD were similar to those in the overall cohort before subgroup analysis. This stability may indicate a critical point for renal injury due to metabolic imbalance, which is influenced by renal lipid metabolism capacity. Instead of changing this threshold, ASCVD likely exacerbates renal damage through inflammation and endothelial dysfunction. Additionally, the larger sample size of diabetic patients without ASCVD may limit the detection of differences in thresholds despite reflecting real-world distributions. Therefore, current observations on threshold consistency should be interpreted cautiously with respect to statistical power and clinical context, and subtle differences might go unnoticed owing to sample size limitations. Furthermore, the loss of the protective effect of HDL-C in ASCVD patients may be caused by functional impairment. The activity of myeloperoxidase (MPO), a proinflammatory oxidase, diminishes the antioxidant capacity of HDL-C. In atherosclerosis, increased serum MPO activity leads to oxidative modification of HDL-C (26, 27), potentially reducing its renoprotective function.

Interaction analysis revealed that ASCVD modifies the relationship between blood lipids and DKD risk in type 2 diabetic patients, highlighting complex interactions between dyslipidemia and renal injury. ASCVD significantly enhances the nephrotoxic effects of TC and LDL-C, with both multiplicative and additive interactions showing statistical significance. The presence of these two interactions represents a strong form of interaction (28). The additive interaction results indicate that lowering LDL-C and TC may reduce DKD risk more effectively in ASCVD patients than in those without ASCVD. The inconsistent multiplicative interaction among different gender and age populations indicates that at the relative risk level, ASCVD does not significantly change the intensity of the effect ratio of blood lipids on the risk of DKD. However, the additive interaction among people of different genders and ages is significant, which has important public health significance, especially when assessing the hazards of combined exposure. It indicates that simultaneous exposure to two factors can lead to an excessive increase in risk, which may be more dangerous than exposure alone.

ASCVD amplifies lipid-driven renal injury through synergistic pathways:systemic inflammation promotes LDL oxidation to pro-atherogenic ox-LDL (29, 30), activating pro-fibrotic TGF-β signaling in glomerular cells. Endothelial dysfunction impairs renal vascular integrity, enhancing subendothelial retention and oxidation of atherogenic lipoproteins like oxidized LDL (31, 32), which directly induce podocyte injury, mesangial cell apoptosis, and tubular epithelial cell damage via lipid peroxidation and ER stress. Atherosclerotic renal hypoperfusion activates the renin-angiotensin-aldosterone system (RAAS) (33, 34), accelerating proteinuria and tubulointerstitial fibrosis.

Limitations include: first, the cross-sectional design limits causal inference and risks reverse causation. Second, residual confounding from unmeasured variables such as dietary patterns and genetic polymorphisms in lipid metabolism genes like APOE or PCSK9 cannot be ruled out. Such factors may independently modulate systemic lipid exposure and renal lipid metabolism. Lack of medication data (e.g., RAAS inhibitors) and diabetes duration may introduce residual confounding. Future studies should incorporate these variables. Future research should validate lipid thresholds through multicenter studies, assess the impact of threshold-directed lipid management on DKD incidence via interventional trials, and define optimal lipid target ranges. Additionally, mechanistic studies should clarify ASCVD-lipid metabolism interactions, especially concerning oxidative stress and RAAS pathways, for comprehensive pathophysiological insights. Despite these limitations, this work provides significant evidence for early DKD prevention by emphasizing nonlinear risk patterns and ASCVD in lipid management. It shifts lipid management from universal numerical targets to precision threshold-based approaches, ultimately facilitating personalized lipid control and organ-specific protection.

## 5 Conclusion

This research explores the link between blood lipids and DKD risk, highlighting the effect of ASCVD on these associations. Key findings indicate that in diabetic patients with ASCVD, LDL-C and TC were linearly related to DKD risk; and only TG exhibited a significant nonlinear association. In contrast, diabetic patients without ASCVD displayed significant nonlinear threshold effects for all four lipids on DKD risk. Importantly, interaction analysis revealed that ASCVD accelerated the nephrotoxic effects of TC and LDL-C. These results emphasize distinct clinical implications: diabetic patients with ASCVD need strict control of TC and LDL-C levels to reduce DKD risk, whereas those without ASCVD benefit from threshold-based lipid management. This work provides essential evidence for personalized clinical decision-making and suggests the need for future research into synergistic ASCVD-lipid mechanisms and targeted therapies.

## Data Availability

The raw data supporting the conclusions of this article will be made available by the authors, without undue reservation.
