# Supplementary material for: The interaction between blood lipids and ASCVD increases the risk of DKD: a nonlinear relationship transforms into a linear relationship, a cross-sectional study
Source: Front Endocrinol (Lausanne). 2025 Aug 20;16:1652396. doi: 10.3389/fendo.2025.1652396 (PMC12405954; doi:10.3389/fendo.2025.1652396)
Supplement: Supplementary file 1 [file Table1.docx]

Supplementary Table 1 Association of Blood Lipids with DKD Risk by ASCVD (Stratified by gender and age）

| blood lipid compenent | with ASCVD | OR | 95%CI Lower Limit | 95%CI Upper Limit | Wald value | p value | p value of multiplicative interaction |
| --- | --- | --- | --- | --- | --- | --- | --- |
| Male |  |  |  |  |  |  |  |
| TG | no | 1.040 | 1.013 | 1.065 | 3.116 | 0.002 | 0.679 |
|  | yes | 1.041 | 0.954 | 1.154 | 0.835 | 0.404 |  |
| TC | no | 1.060 | 0.996 | 1.124 | 1.870 | 0.062 | 0.01199 |
|  | yes | 1.271 | 1.084 | 1.500 | 2.890 | 0.004 |  |
| HDL-C | no | 0.373 | 0.274 | 0.505 | -6.304 | <0.001 | 0.7736 |
|  | yes | 0.421 | 0.209 | 0.846 | -2.434 | 0.015 |  |
| LDL-C | no | 0.998 | 0.919 | 1.083 | -0.040 | 0.968 | 0.000855 |
|  | yes | 1.384 | 1.137 | 1.696 | 3.192 | 0.001 |  |
| Female |  |  |  |  |  |  |  |
| TG | no | 1.057 | 1.012 | 1.098 | 2.671 | 0.008 | 0.6578 |
|  | yes | 1.058 | 0.949 | 1.248 | 0.805 | 0.421 |  |
| TC | no | 1.160 | 1.073 | 1.251 | 3.797 | <0.001 | 0.1085 |
|  | yes | 1.407 | 1.105 | 1.829 | 2.654 | 0.008 |  |
| HDL-C | no | 0.578 | 0.415 | 0.800 | -3.264 | 0.001 | 0.1767 |
|  | yes | 1.342 | 0.542 | 3.417 | 0.631 | 0.528 |  |
| LDL-C | no | 1.125 | 1.020 | 1.240 | 2.374 | 0.018 | 0.2779 |
|  | yes | 1.321 | 0.997 | 1.780 | 1.888 | 0.059 |  |
| Age>=60 |  |  |  |  |  |  |  |
| TG | no | 1.042 | 0.989 | 1.090 | 1.683 | 0.092 | 0.2686 |
|  | yes | 1.109 | 0.968 | 1.314 | 1.334 | 0.182 |  |
| TC | no | 0.998 | 0.918 | 1.084 | -0.043 | 0.966 | 0.002234 |
|  | yes | 1.349 | 1.120 | 1.637 | 3.099 | 0.002 |  |
| HDL-C | no | 0.595 | 0.436 | 0.808 | -3.290 | 0.001 | 0.6041 |
|  | yes | 0.738 | 0.361 | 1.515 | -0.832 | 0.405 |  |
| LDL-C | no | 0.959 | 0.868 | 1.058 | -0.832 | 0.405 | 0.003642 |
|  | yes | 1.359 | 1.091 | 1.707 | 2.690 | 0.007 |  |
| Age<60 |  |  |  |  |  |  |  |
| TG | no | 1.042 | 1.016 | 1.066 | 3.421 | 0.001 | 0.705 |
|  | yes | 1.018 | 0.945 | 1.122 | 0.423 | 0.672 |  |
| TC | no | 1.138 | 1.076 | 1.201 | 4.641 | <0.001 | 0.1998 |
|  | yes | 1.261 | 1.046 | 1.541 | 2.341 | 0.019 |  |
| HDL-C | no | 0.349 | 0.251 | 0.482 | -6.323 | <0.001 | 0.2687 |
|  | yes | 0.542 | 0.227 | 1.310 | -1.374 | 0.170 |  |
| LDL-C | no | 1.111 | 1.024 | 1.204 | 2.543 | 0.011 | 0.07077 |
|  | yes | 1.356 | 1.072 | 1.737 | 2.474 | 0.013 |  |

Supplementary Table 2 Effects of the additive interaction of lipids and ASCVD on the risk of DKD(Stratified by gender and age）

|  | RERI(95%CI) | AP(95%CI) | SI(95%CI) |
| --- | --- | --- | --- |
| Male |  |  |  |
| TG | 2.79(-1.47,7.05) | 0.06(-0.03,0.14) | 1.06(0.96,1.17) |
| TC | 6.46(4.33,8.60)*** | 0.23(0.11,0.35)*** | 1.32(1.11,1.56)*** |
| HDL-C | -24.22(-55.17,6.72) | -1.32(-2.95,0.30) | 0.42(0.20,0.85) |
| LDL-C | 9.15(5.82,12.48)*** | 0.29(0.15,0.43)*** | 1.44(1.16,1.77)*** |
| Female |  |  |  |
| TG | 4.38(-2.30,11.06) | 0.08(-0.05,0.21) | 1.09(0.94,1.26) |
| TC | 9.60(4.82,14.38)*** | 0.28(0.12,0.45)*** | 1.41(1.10,1.81)*** |
| HDL-C | 3.58(-18.55,25.71) | 0.13(-0.66,0.91) | 1.15(0.45,2.94) |
| LDL-C | 11.35(4.20,18.50)*** | 0.23(0.03,0.43)* | 1.31(0.99,1.72)* |
| Age>=60 |  |  |  |
| TG | 5.02(-0.35,10.38) | 0.12(-0.02,0.26) | 1.14(0.97,1.34) |
| TC | 4.53(2.73,6.34)*** | 0.26(0.12,0.39)*** | 1.37(1.12,1.69)*** |
| HDL-C | -8.93(-33.18,15.32) | -0.35(-1.29,0.58) | 0.73(0.36,1.49) |
| LDL-C | 6.92(3.65,10.20)*** | 0.26(0.10,0.42)*** | 1.37(1.08,1.72)*** |
| Age<60 |  |  |  |
| TG | 1.55(-3.57,6.67) | 0.02(-0.06,0.10) | 1.02(0.94,1.11) |
| TC | 10.82(6.87,14.77)*** | 0.22(0.08,0.37)*** | 1.29(1.06,1.57)* |
| HDL-C | -14.47(-45.69,16.74) | -0.70(-2.18,0.78) | 0.58(0.24,1.42) |
| LDL-C | 14.27(8.00,20.53)*** | 0.28(0.11,0.45)*** | 1.40(1.09,1.79)*** |

****p* value <0.001,**p* value <0.05
